# Legitimacy of investigative forensic genetic genealogy under Art. 8 ECHR

**DOI:** 10.1016/j.fsisyn.2025.100636

**Published:** 2025-08-18

**Authors:** Oliver M. Tuazon, Bart Custers, Gerrit-Jan Zwenne

**Affiliations:** Center for Law and Digital Technologies (eLaw), Institute for the Interdisciplinary Study of the Law, Leiden Law School, Leiden University, Kamerlingh Onnes Building, Steenschuur 25, Leiden, 2311 ES, the Netherlands

**Keywords:** Investigative forensic genetic genealogy (iFGG), European Convention on Human Rights (ECHR), European Court of Human Rights (ECtHR), ECtHR four-fold privacy test, Genetic and genometric data privacy, Forensics, DNA

## Abstract

Investigative forensic genetic genealogy (iFGG) was successfully used in the United States to solve the Golden State Killer case in 2018 and in Sweden to solve the Linköping double-murder case in 2020. However, further use of iFGG in Sweden was temporarily suspended due to concerns about its legitimacy. This article evaluates the legitimacy of iFGG within what we name, the European Court of Human Rights' (ECtHR) four-fold privacy test: the preliminary interference test, the lawfulness test, the legitimate aim test, and the proportionality test. The use of iFGG is an interference with an individual's right to respect for private life under Article 8 of the European Convention on Human Rights (ECHR). Its lawfulness requires the creation of an iFGG-enabling law or amendment of an existing law to allow iFGG use by law enforcement. Its legitimate aim—criminal identification through data derived from DNA deposited at crime scenes—falls squarely under Article 8 § 2 ECHR. The proportionality of its use largely depends on the provision of appropriate safeguards in an iFGG-enabling law that would protect genetic data privacy. Although iFGG is a powerful tool to help solve cold cases, it has to stand on a solid legal ground that allows its use while respecting the right to privacy. It should be able to withstand any legal challenge before the ECtHR in the future. The safeguards identified in this article, if incorporated in an iFGG-enabling law, hope to prevent such legal challenge.

## 1 Introduction

Investigative forensic genetic genealogy (iFGG) is a relatively new criminal identification technique [[1], [2], [3]], whose nomenclature, definition and scope we previously clarified [4]. It has been successfully used to solve cold cases in the United States, the most famous of which is the Golden State Killer case in 2018 [1,5]. In Europe, pilot studies have been carried out in Sweden and Norway [6,7], and a Dutch court has allowed its use in the Netherlands [[8], [9], [10]].

The use of iFGG in solving cold cases is nevertheless received with both approval and criticism [2,11,12]. On the one hand, perpetrators of decades-long unresolved cases are finally brought to courts and the families of their victims find closure. On the other hand, questions of privacy arose, in particular, the right to privacy with respect to genetic data stored in commercial DNA databases and that of their un-enrolled genetic relatives who unwittingly became involved in criminal investigations [13].

Aside from technical issues, such as the hacking of a commercial database that potentially exposed the personal data of users who opted out of law enforcement searches [14,15], a big cause of concern is the personal data that is potentially revealed by the use of iFGG. Moreover, besides its ability to trace one's genetic relatives, the promise of predicting one's medical predispositions by some DTC-GT companies has caught the imagination of millions of their subscribers [16]. It is not clear, however, how many of these consumers realize that they are sharing their most intimate biological data: their DNA [17]. While it is possible to change one's name, home address, password, or other personal data, genetic identity is biologically-embedded, rendering it, in effect, individually immutable. More so, it is not clear whether consumers of these commercial databases, who may have (consciously or unknowingly) opted in for law enforcement use of their data, understand clearly that they can become conduits to the possible identification of a relative, near or distant, who may be implicated in a crime.

The debate continues and is largely unresolved, especially in Europe. What is clear is that the use of iFGG should be subject to appropriate safeguards that protect the right to data privacy while law enforcement conducts its legitimate role of solving crime [18,19]. In order to promote “reasoned exercise” in the use of iFGG in the United States—the country which pioneered iFGG—its Department of Justice issued an interim policy that provides guidance on its use to protect “reasonable interests in privacy” (p. 1) [20]. At the level of the Council of Europe (CoE), there is no similar regulation concerning iFGG. However, law enforcement use of genetic data—albeit in the context of law enforcement DNA databases—has already been a subject of case law at the European Court of Human Rights (ECtHR), from the admissibility case of *Van der Velden v. the Netherlands* in 2006 [21], the landmark Grand Chamber case of *S. and Marper v. the United Kingdom* in 2008 [22], up to the more recent case of *Petrović v. Serbia* in 2020 [23]. In all these cases using short tandem repeat (STR) data, the ECtHR has classified these data as protected data under Article 8 of the European Convention on Human Rights (ECHR) or the right to respect for private life [24]. A future case before the ECtHR involving single nucleotide polymorphism (SNP) data using iFGG is expected to fall under the same classification arguably with its own nuances.

In this light, a report on the feasibility of the use of iFGG in the UK—a state signatory of the ECHR—concluded that the “legality and necessity of police use of genetic genealogy (and associated interference with privacy) would need to be clearly established, with reference to Article 8 of the European Convention of Human Rights (ECHR)” (p. 13) [25]. This article takes off from this recommendation. It sees iFGG as one of those “novel ways” which may adversely affect our “private-life interests”, as the ECtHR has forewarned in *Marper* (para. 71) [22]. Keeping in mind ECtHR's tendency to find a middle ground between the right to one's privacy on the one hand, and society's interest in solving crimes on the other hand, this article does not *prima facie* reject the use of iFGG. It rather seeks appropriate safeguards for its use in Europe mainly using ECtHR jurisprudence on law enforcement use of DNA data as our guide given the scope of this article. We earlier coined the term ECtHR's four-fold privacy test (cf. section 2) [26] and applied it in this article to assess the legitimacy of iFGG within the ECHR regime.

This article is divided into seven sections. Section 2 provides a short overview of the ECtHR four-fold privacy test. Sections 3, 4, 5, 6 then cover each component of the four-fold test as applied to iFGG. Section 3 covers the preliminary interference test, on whether the use of iFGG is an interference with private life. Section 4 covers the lawfulness test, on whether the use of iFGG is lawful under the prevailing legal instruments of the Council of Europe. Section 5 covers the legitimate aim test, on whether the use of iFGG would serve any legitimate purpose under Article 8 § 2 ECHR. Section 6 covers the proportionality test, on whether the use of iFGG is proportional to the legitimate purpose it purports to serve. As it goes with the ECtHR's Article 8 jurisprudence, the bulk of the discussion on appropriate safeguards is found in this section and the safeguards identified in the other tests find their full significance when discussed under the lens of proportionality. Section 7 puts together the various conclusions of the ECtHR four-fold privacy test and provides an answer on the legitimacy of iFGG within the ECHR regime.

## 2 The ECtHR four-fold privacy test: a quick overview

The concept of private life under Art. 8 ECHR has a wide scope and is “not susceptible to exhaustive definition” (para. 36 [27]) [28,29]. In adjudicating cases that fall under the right to respect for private life under Article 8 ECHR, the ECtHR balances two key interests: an applicants' claim that their right to privacy has been breached—in this case through the collection and retention of their DNA data—and the government's or society's legitimate interest in solving crimes through the use of DNA data [13]. There are previous studies focusing on the bioethical, relational and social aspects of the use of DNA data [12,[30], [31], [32], [33], [34]], including more recently, on genometric data privacy [13]. The focus of this article is on assessing the legitimacy of iFGG, which uses DNA data, within the ECHR regime following ECtHR jurisprudence.

The ECtHR follows a method in assessing the legitimacy of any technique or method that may affect the right to privacy under Article 8 ECHR. We earlier coined the term *ECtHR four-fold right to privacy test* to refer to this method (p. 24) [26]. In this article, we also refer to its short form, the *ECtHR four-fold privacy test*, or simply, the *four-fold test*. Although the ECtHR never mentioned that term directly in its jurisprudence, it can be derived from Article 8 § 2 ECHR and from ECtHR case law, such as in *S. and Marper v. the United Kingdom* (paras. 59–86, 95–124 [22]), *Peruzzo and Martens v. Germany* (paras. 32–49 [35]), and *Gaughran v. the United Kingdom* (paras. 63–86 [36]).

We have previously applied the four-fold test in assessing the acceptability of universal forensic DNA databases within the ECHR regime [37]. The components of the four-fold test, without naming it as such, were also present in the evaluation of the acceptability of another DNA-based technique, forensic DNA phenotyping, in Europe (p. 11) [38]. In this article, we apply it to iFGG. In short, the ECtHR initially evaluates the presence of an interference against a protected right (preliminary interference test); if affirmative, the ECtHR checks if the interference has a basis in law (legality test), whether its purpose is covered by Article 8 § 2 ECHR (legitimate aim test) and whether it is necessary in a democratic society within the margin of appreciation afforded to the member-state party under litigation (necessity or proportionality test).

## 3 The preliminary interference test

The preliminary interference test is the initial evaluation of the legitimacy of iFGG within the Article 8 ECHR regime, specifically, on whether law enforcement's use of DNA data in criminal investigations constitutes an interference with an individual's private life. The presence of an interference does not automatically mean a violation of Article 8 ECHR. The ECtHR has to proceed with the three other components of the four-fold test before making a final declaration of a violation (sections 4, 5, 6).

### 3.1 SNP profile data: protected under Article 8 ECHR

The ECtHR cases involving DNA data use by law enforcement in criminal investigations—*Van der Velden v. the Netherlands* [21], *S. and Marper v. the United Kingdom* [22], *W. v. the Netherlands* [39], *Peruzzo and Martens v. Germany* [35], *Aycaguer v. France* [40], *Gaughran v. the United Kingdom* [36], *Trajkovski and Chipovski v. North Macedonia* [41], and *Dragan Petrović v. Serbia* [23]—were in the form of short tandem repeat (STR) profiles. In all these cases, the ECtHR classified DNA data as protected data under Article 8 ECHR. On the other hand, iFGG uses DNA data in the form of single-nucleotide polymorphism (SNP) profiles.

STR profiles reveal less personal data compared to SNP profiles [4,42], although recent research on STRs shows that some of the markers may reveal some traits that were not known before [[43], [44], [45]]. STR profiles involve less than 30 markers whereas SNP profiles used by DTC-GT companies usually use more than 600,000 markers [2,3,20]. And these SNP markers have the potential to reveal more sensitive personal information such as propensity to disease and other health markers [5]. It is for this reason why pharmaceutical companies are interested in purchasing these sensitive data [46,47].

Given these considerations, it would be easy to second guess the ECtHR's prospective classification of SNP profile data as protected data under the ECHR regime. If the ECtHR has classified STR profiles as protected data, *a fortiori*, it would also classify SNP profiles, which can potentially reveal more personal information, as protected data. Hence, following existing case law [[21], [22], [23],^35^,^36^,[39], [40], [41]], DNA data, both in the form of STR or SNP profiles, can be considered protected data under the ECHR regime.

The prospective classification of SNP profile data as protected data under the ECHR Article 8 regime may also be based on an *obiter dictum* of the ECtHR in *Marper*: “That only a limited part of this information is actually extracted or used by the authorities through DNA profiling and that no immediate detriment is caused in a particular case does not change this conclusion.” (para. 73) [22]. That statement shows that the protection of DNA data by the ECtHR is “technology proof”, i.e., it does not depend on whether future scientific breakthroughs would discover a technique that will only process a few DNA markers in order to be able to identify its individual source (p. 16) [13]. This is sufficient reason to support the presence of an interference when law enforcement employs SNP data in iFGG, aside from the fact that it contains more sensitive personal data—hence, more privacy intrusive—compared to STR data.

### 3.2 Preliminary safeguard: iFGG as a method of last resort

Given that SNP data used in iFGG potentially reveals more sensitive data compared to STR data as discussed in section 3.1, an appropriate safeguard is to allow law enforcement to use it in criminal investigations as a method of last resort. The ECtHR recognized in *Marper* the power of DNA data to identify genetic relatives, aside from the sensitive personal data that it can potentially reveal (paras. 39 & 72) [22]. This recognition applies to a greater extent in iFGG, as shown in the Golden State Killer who was not identified using STR-based methods. The latter's use in familial searching is limited to close relatives such as “parents/offspring and full sibling” (p. 2 [4]) [5]. The GSK was identified via iFGG with different sources claiming that it was done through a second, third, or even a probable fourth cousin [1,48,49]. However, as a method of last resort—despite SNP data's greater potential over STR data—law enforcement should not be allowed to use it at the commencement of a criminal investigation. This safeguard is based on the data minimization principle, specifically, that law enforcement should only use iFGG—which makes use of SNP data that potentially expose more sensitive data—after using STR-based methods without producing a suspect lead. It provides a preliminary layer of protection against the hasty use of iFGG as was initially suspected in the Idaho quadruple murder case involving Bryan Kohberger in 2022, which allowed his defense lawyers to request the court for a copy of evidence that the prosecution withheld [50]. It was eventually shown that law enforcement indeed used STR data first but later accessed restricted commercial genetic genealogy databases for iFGG [51].

This is only a preliminary safeguard given that it flows from the discussion on the SNP v. STR profile data in section 3.1, i.e., law enforcement should only resort to iFGG which makes use of SNP data after the STR-based methods—both of which are considered interferences to a person's private life when used in criminal investigations under Article 8 ECHR—did not yield a suspect lead. iFGG as a method of last resort is expounded further and more relevantly under the proportionality test when we discuss the factors from previous ECtHR case law which presuppose iFGG as a method of last resort (cf. section 6.2) and the safeguards related to the iFGG process which procedurally shows that STR-based methods have to be employed first and found wanting before resorting to iFGG (cf. section 6.3).

## 4 The lawfulness test

The second component of the four-fold test is the lawfulness or legality test. Under this test, two major requirements are assessed by the ECtHR. *First*, the measure under consideration should have a legal basis in domestic law (section 4.1). For the purposes of this article, we evaluate the CoE legal instruments cited by the ECtHR in its case law involving law enforcement use of DNA data [[21], [22], [23],^35^,^36^,[39], [40], [41]] (section 4.1). *Second*, the applicable law should pass a certain quality standard [52,53] (section 4.2).

### 4.1 DNA-related laws at the Council of Europe

iFGG makes use of DNA-SNP data, from which genetic associations are derived leading to the generation of a suspect investigational lead [1,2,4]. We present legal instruments at the CoE level covering DNA data, most of which have been cited in ECtHR case law involving law enforcement use of DNA data [[21], [22], [23],^35^,^36^,[39], [40], [41]]. The main legislative basis of the four-fold privacy test is Article 8 § 2 ECHR and these legal instruments are only supplementary to it [24].

The first European-wide data protection law was the CoE Data Protection Convention (Convention 108) [54]. However, it was the EU General Data Protection Regulation (GDPR) that first defined the term genetic data [55]. A couple of years later, Convention 108 was updated into Convention 108+ where the term *genetic data* was defined (art. 6 [18]; no. 57 [19]). To date, it only has 33 out of 38 ratifications needed for it to enter into force [56]. However, we cite Convention 108+ given the tendency of the ECtHR to even consider “unwritten” law in its evaluation (paras. 28 & 29 [57]) [58]. At any rate, both Convention 108 and Convention 108+ do not prevent the use of SNP data in criminal investigations. They only require the provision of appropriate safeguards to protect the rights and freedoms of data subjects (art. 6) [18,54].

The Convention on Human Rights and Biomedicine (Oviedo Convention), was not mentioned in the ECtHR case law cited in section 3.1. It may be because it is a convention on biomedicine. However, it contains a chapter on “Private life and right to information” where everyone's “right to respect for private life in relation to information about his or her health” was included (Article 10 (1) [59]). This is relevant because the ECtHR in *Marper* was concerned about the “highly personal nature of cellular samples” which contain sensitive information about an individual's health (para. 72) [22]. One may argue that the respondent state in *Marper* (UK) has not ratified the Oviedo Convention, hence, it does not apply to it. However, other member states cited in the case list in section 3.1., such as France, North Macedonia and Serbia ratified the Oviedo Convention [60]. The omission of the Oviedo Convention from the list of legislation discussed in previous ECtHR case law involving law enforcement use of DNA data may support the argument that the focus of the ECtHR in these cases is DNA data for criminal identification, not for the elaboration of their medical or other traits.

Recommendation No. R (87) 15 of the Committee of Ministers (Police Recommendation) concerns the regulation of the use of personal data in the police sector [61]. It did not cover genetic data specifically, but the use of personal data in general whenever processed by the police. In a later practical guide on the Police Recommendation, a definition of genetic data was included (p. 18) [62]. Principle 2.1 requires that the collection of personal data is “limited to such as is necessary for the prevention of a real danger or the suppression of a specific criminal offence”. Principle 3.1 limits the storage of personal data to “accurate data and to such data as are necessary to allow police bodies to perform their lawful tasks”. Principle 7.1 requires the deletion of these stored personal data “if they are no longer necessary for the purposes for which they were stored”. These three specific requirements of the Police Recommendation apply to DNA data used in iFGG. They do not necessarily prohibit the use of iFGG but they can serve as part of the safeguards of its enabling law (cf. section 4.3).

Whereas the Police Recommendation covers personal data in general [61], Recommendation No. R (92) 1 specifically covers the use of DNA data within the framework of the criminal justice system [63]. Provision 8 is similar to Principles 3 and 7 of the Police Recommendation, although applied more specifically to DNA-related data. It provides that cellular samples collected for DNA analysis should be discarded after the final decision of the case “unless it is necessary for purposes directly linked to those for which they were collected”. It provides a window for a longer retention period for “serious offences against the life, integrity or security of persons”. But in any case, the storage period should be defined by law. None of the provisions of Recommendation No. R (92) 1 prohibit the use of iFGG in criminal investigations.

Recommendation No. R (97) 5 of the Committee of Ministers was the first CoE Recommendation that defined the term *genetic data*, almost a decade before it was defined in EU's GDPR (provision 1) [64]. It was updated and replaced by Recommendation CM/Rec (2019) 2 [65]. Provision 7.3 clarified the apparent prohibition in the old version of the law on the use of “other characteristics”—which would have affected iFGG use given that it may reveal many characteristics—as long as “appropriate safeguards are provided for by law” [38,65].

In *Marper*, the ECtHR also made a quick survey of practices in CoE member states on the collection and retention of DNA data (paras. 107–112) [22]. These practices were evaluated again 12 years later in *Gaughran* (paras. 81–84) [36]. Although the ECtHR noted a variety of storage regimes—even with a minority having indefinite retention regimes (para. 82) [36]—the Court adjudged that there is still a “consensus” among CoE member states as to following the storage limitation principle (para. 112 [22]; para. 84 [36]). As discussed in section 6.1, such a consensus narrows the margin of limitation afforded to CoE member states.

### 4.2 Quality of DNA-related laws

The second aspect of the lawfulness test is the assessment of the quality of the law, presumably in the case at hand, a new CoE Recommendation that will serve as a template for domestic iFGG-enabling laws (cf. section 4.3). Given that such a Recommendation does not exist yet, the discussion in this section is limited to the quality of the CoE legal instruments discussed in section 4.1. The ECtHR also has a more expansive view of the meaning of law, following its “substantive” and not its “formal” sense, even including “both enactments of lower rank than statutes and unwritten law” (paras. 28 & 29 [57]) [58]. In any case, any legal basis should be accessible to the people covered by the law, foreseeable as to its consequences and sufficiently clear [22,52,53].

Accessibility requires that the public is made aware of the law's existence through promulgation and publication [58]. The question of accessibility of the law has not been raised before the ECtHR in the context of genetic data. What is usually questioned is the sufficiency of the enabling law that was applied by law enforcement to justify their use of DNA data. In the case of *Petrovic*, for example, the ECtHR considered that the legal basis used by the police—Article 131 §§ 2 and 3 of the Code of Criminal Procedure of the concerned member state—was insufficiently applied in taking DNA samples from the applicant [23]. The final verdict of the ECtHR was nailed by the law's lack of foreseeability.

Foreseeability means that the law should be “formulated with sufficient precision to enable the individual—if need be with appropriate advice—to regulate his conduct” (para. 95) [22]. In *Petrovic*, the ECtHR took issue with the lack of “specific reference” in the law to the “taking of a DNA sample” (para. 81) [23]. As applied to iFGG, the meticulousness exhibited by the ECtHR in *Petrovic* highlights the need for a specific law allowing the use of iFGG by law enforcement in criminal investigations (cf. section 4.3).

As to clarity of the law, the ECtHR explained that the law “must afford adequate legal protection against arbitrariness and accordingly indicate with sufficient clarity the scope of discretion conferred on the competent authorities and the manner of its exercise” (para. 95) [22]. In *Petrovic*, the ECtHR sided with the applicant who claimed that the provision in the law used by the police in taking his DNA sample—“other medical procedures”—was “too vague” to be applied to his case (para. 62) [23]. However, one judge dissented claiming, “How can the words ‘other medical procedures’ be construed not to encompass this type of evidence?” [66] In order to avoid possible variance in its interpretation, the iFGG-enabling law should be sufficiently clear as to the types of samples covered and their subsequent processing for criminal identification.

### 4.3 Safeguard: a CoE Recommendation on iFGG

At the CoE level, the Committee of Ministers issues Recommendations that are “often precursors of legally binding agreements, testing the ground and helping to shape the consensus that may eventually lead to directly enforceable European standards” (para. 12) [67]. Following the qualities of foreseeability and clarity (cf. section 4.2), the Committee of Ministers could consider issuing a Recommendation on iFGG use by law enforcement within the CoE. This legal safeguard would serve as a guide or legal template to harmonize iFGG-enabling domestic laws among CoE member states, thereby facilitating inter-member state cooperation in criminal investigations. For its implementation within a CoE member state, it is imperative that iFGG use also has a legal basis, i.e., an iFGG-enabling law, which can be in the form of a separate law on iFGG, or an existing law with specific provisions allowing iFGG use like in the case of Sweden and Denmark [[68], [69], [70], [71], [72]].

We provide three reasons to justify such issuance as a legal safeguard. *First*, a CoE Recommendation would provide a specific legal basis for the use of iFGG among the CoE member states. This is highlighted by the Swedish case. In spite of iFGG's success in solving the Linköping double-murder case, the Swedish Authority for Privacy Protection prevented its further use, mainly due to the absence of a specific iFGG law allowing its use for criminal investigations. Reminiscent of *Petrovic*, the applicable Swedish laws at that time were found insufficient to continue carrying out criminal investigations using iFGG [6,73]. *Second*, a CoE Recommendation would serve as a starting point—a *testing ground*, so to speak—that may help shape consensus among its member states “that may eventually lead to directly enforceable European standards” (para. 12) [67]. In the United States, the Department of Justice issued an Interim Policy for the use of iFGG across the country in 2019. It aimed to “promote the reasoned exercise of investigative, scientific, and prosecutorial discretion” in the use of iFGG in solving criminal cases (p. 1) [20]. An iFGG CoE Recommendation could serve as a legal template that contains the minimum safeguards that should be included in an iFGG-enabling domestic law. An iFGG legal template is already available in the US [74,75]. In the US, Maryland, Montana, Utah and Florida have introduced legislation regulating the use of iFGG for criminal investigations [[76], [77], [78], [79], [80]]. In Europe, Sweden and Denmark have introduced similar legislation that took effect in 2025. An iFGG CoE Recommendation will help in the harmonization of these laws to facilitate inter-state cooperation in criminal investigations using iFGG. *Third*, an iFGG CoE Recommendation would serve as a more pro-active step on the part of the Council of Ministers to fulfill a seeming positive legal obligation to ensure the protection of the genetic data privacy when the member states' law enforcement officials implement iFGG [81,82]. The Swedish case suggests that a positive obligation exists to adopt specific measures to protect data privacy when using iFGG, and for this reason, they have updated their current biometrics law to allow iFGG use in the country [[68], [69], [70]].

## 5 The legitimate aim test

Following the *ratio decidendi* of ECtHR case law, the legitimate aim or purpose test is the most simple and straightforward and it has been the practice of ECtHR “to be quite succinct when it verifies the existence of a legitimate aim” under Article 8 § 2 ECHR (para. 25) [83]. A case in point: In the cases cited in section 3.1, the ECtHR devoted only one paragraph to discuss whether they passed this test. Concretely, iFGG only has to be classified among the legitimate purposes laid down under Article 8 § 2 ECHR [24]. This section proposes a nuanced approach to the legitimate aim test by making a distinction between the mediate and immediate purposes of iFGG (section 5.1) and proposes an appropriate safeguard under this test (section 5.2).

### 5.1 The immediate and mediate purposes of iFGG

In *Marper*, the ECtHR distinguished two purposes of DNA data, which we classify as the immediate purpose (“the original taking of [.] information pursues the aim of linking a particular person to the particular crime”) and the mediate purpose (“its retention pursues the broader purpose of assisting in the identification of future offenders”) of criminal identification (para. 100) [22]. As applied to iFGG, the main difference between these two purposes concerns the subject of criminal identification: the criminal suspect who left the forensic sample in an ongoing investigation (immediate purpose) and future criminal suspects in future investigations (mediate purpose).

The immediate purpose of iFGG—identification of the individual or individuals who left forensic samples at the crime scene—falls squarely under the legitimate purpose of crime prevention under Article 8 § 2 ECHR (para. 100) [22]. As explained in *Van der Velden*, this purpose is “not altered by the fact that DNA played no role in the investigation and trial of the offences committed by the applicant” [21]. We then argue that the ECtHR has provided a justification for the use of iFGG, whose final result—the generation of suspect investigational leads—does not usually end up being used in a court trial [2,4].

The potential mediate purpose of iFGG—identification of future criminal offenders—may be justified by the ECtHR's claim that the same DNA data used in identifying the source may be used for the “broader purpose of assisting in the identification of future offenders” (para. 100 [22]) [36,41]. However, the cited case law refer to STR data, which are less privacy intrusive compared to SNP data. Allowing this potential mediate purpose of iFGG would mean storing SNP data generated from forensic samples, including those from cases that have been solved, which calls for a safeguard under this test.

### 5.2 Safeguard: prohibit the use of iFGG's mediate purpose

There are benefits to storing SNP data in perpetuity by law enforcement—even after the resolution of the case—given their potential to help create genetic links to samples from other cases they are solving or will solve in the future. However, the *legitimacy* of that practice is difficult to justify under the current ECHR regime because the use of SNP data to identify other criminal perpetrators in other investigations goes beyond the purpose of its collection—i.e., the identification of the forensic source (immediate purpose)—thereby violating the purpose limitation principle. Hence, an appropriate safeguard is to limit the use of iFGG to its immediate purpose and to prohibit the use of its potential mediate purpose. This means that SNP data should be destroyed once the case is solved with finality, that is, without the possibility of further appeal. The current ECtHR case law (cf. section 3.1) refer to known individuals' STR data which contain less sensitive personal data compared to SNP data, and the Court already restricted the retention periods of these data. We then opine that the ECtHR will be less disposed to allow the storage of SNP data, even from forensic samples, once their purpose of identifying the source has been achieved, that is, once the SNP data are already linked to a known individual with finality. Besides, the iFGG process only produces suspect leads, whose identification would still have to be confirmed by standard STR methods [[1], [2], [3]]. And it is these STR data that are stored in law enforcement DNA databases, whose retention is also aimed at identifying future offenders (para. 100) [22]. Moreover, the retention of SNP data for iFGG's mediate purpose is not practical given that it would entail additional cost of building a separate database, whereas it is expected to only retain a few profiles given the preliminary safeguard of iFGG as a method of last resort (cf. section 3.2).

This safeguard is bolstered by the fact that the Linköping case—the first application of iFGG in Europe—was solved without the presence of previous SNP data stored by law enforcement [6,73]. It then shows that there is no urgency in retaining SNP data of solved cases when the same goal of criminal identification can be achieved with less data-privacy intrusive measures. This safeguard is also in line with Article 5 (4e) of Convention 108+ on the lawful processing of personal data, which should not be kept “longer than is necessary for the purposes for which those data are processed” [18]. One may argue that the purpose can be extended by the iFGG-enabling law (cf. section 4.3) to the resolution of other and future crimes (mediate purpose). In this way, the SNP generated data will not be wasted through deletion and it can be used for future crime solving. However, the ECtHR's current predisposition will not allow perpetual retention of these sensitive data not only for the unconvicted as shown in *Marper* [22] but also for the convicted as shown in *Gaughran* [36].

We understand that in some jurisdictions—where “perpetrators who discard DNA at crime scenes have no presumed right to privacy” (p.14) [3]—there is more liberality in storing sensitive personal data without time limits, but this article concerns iFGG's application in Europe, which is more protective of data privacy especially when it involves access by law enforcement [[84], [85], [86]]. We do not find any reason for the ECtHR to allow perpetual retention of SNP data even if they come from forensic samples, no matter how useful they may be for future law enforcement searches, not only because of the purpose limitation principle as discussed above but also in keeping with the storage limitation principle that is found not only in Convention 108 (art. 5 (1) (e) [54]) and its modernized version (art. 5 (4) (e) [18]) but also in the GDPR (art. 5 (e) [55]) and in the LED (arts. 4 (1) (e) & 5 [87]).

## 6 The proportionality test

The last component of the ECtHR four-fold test is the proportionality test. Although the word does not appear in the ECHR text, “proportionality is at the heart” of the Court's interpretation of the meaning of the phrase “necessary in a democratic society” under Article 8 § 2 ECHR [88]. This test has been applied in different ways by the ECtHR and it tends to “confuse and mix” various elements which makes the structuring of an analysis quite challenging (p. 467) [89]. We follow the ECtHR's analytical structure where an interference is considered necessary in a democratic society if it answers a “pressing social need”, it is “proportionate to the legitimate aim pursued”, and the reasons to justify it are “relevant and sufficient” (para. 101) [22] (sections 6.1, 6.2). We also analyzed the various steps involved in iFGG and determined safeguards that protect data privacy (section 6.3).

### 6.1 iFGG as a pressing social need

Four factors have been identified to assess the presence of a pressing social need and we apply them to iFGG (p.8) [90]: 1. Is the measure seeking to address an issue which, if left unaddressed, may result in harm to or have some detrimental effect on society or a section of society? 2. Is there any evidence that the measure may mitigate such harm? 3. What are the broader views (societal, historic or political, etc.) of society on the issue in question? 4. Have any specific views/opposition to a measure or issue expressed by society been sufficiently taken into account?

*First*, iFGG seeks to address the issue of crime solving, which “may result in harm to or have some detrimental effect on society” if left unsolved [90]. *Second*, there is evidence that iFGG can solve cold cases. Its fame arose through the identification of the Golden State Killer in the United States [1] and the Linköping murderer in Sweden [6]. *Third,* there is no question that the issue—crime solving—is something that everyone in society desires and everyone feels safer in a society where no one can get away from felonies they commit. The *fourth* factor brings about some difficulty when applied to iFGG because it is not currently accepted by everyone in society. There are people who have *serious reservations* on its use [3], what with reported abuses by genealogists employed by law enforcement, such as those who took advantage of a loophole in the genetic genealogy database to access personal data of consumers who opted out of law enforcement searches [15,91]. At the same time, a recent survey in the US shows that more than 90 % endorse iFGG in solving violent crimes [92]. To put this issue in perspective, it is worth highlighting that the current use of law enforcement DNA databases is also not accepted by everyone [93,94], but DNA evidence has replaced traditional fingerprinting as the gold standard in crime solving [95]. It is then important to understand that the fourth factor does not require complete adherence by everyone in society—an ideal that is almost impossible to achieve in a pluralistic society. What it only requires is that these opposing views are “sufficiently taken into account” [90]. We understand that in the US, there is “no right to privacy for discarded samples” (p. 123) [1]. In Europe, however, the right to privacy of those data subjects—at least once the DNA sample is linked to an individual, regardless of the source—is still acknowledged, although law enforcement may interfere with this right [22,36]. It is the legitimacy of this interference that is precisely being considered in this article. Putting together all the four factors, we can argue that there is a pressing social need to solve cold cases using iFGG.

The ECtHR also connected the assessment of the existence of a *pressing social need* with the margin of appreciation that is afforded to the member state under litigation (para. 59 [96]) [22,35,90]. The reasons adduced by the ECtHR in *Marper* may apply to iFGG for a greater reason since it involves SNP data compared to the more limited STR data. *First*, the DNA data used in iFGG refer to a person's most intimate and unique personal data, which demands that the margin afforded to member states should be narrower [22]. Moreover, the iFGG process takes advantage of genetic relationships to produce suspect investigational leads, which is another factor that narrows the margin of appreciation [36]. *Second*, the survey of DNA-related laws made by the ECtHR in both *Marper* (paras. 107–112) [22] and *Gaughran* (paras. 78–84) [36] may be applied to the DNA data used in iFGG albeit in a stricter fashion. If the ECtHR declared a “strong consensus” among member states in these case law with respect to the use of STR profile data (para. 112) [22], all the more would this consensus be predictably stronger given that the SNP profile data used in iFGG potentially reveal more personal data.

### 6.2 Factors evaluated in previous ECtHR case law

The ECtHR listed various factors to consider in assessing whether the measure at hand is proportional to the legitimate aim pursued (para. 119) [22]. We re-classify these factors according to the peculiarities of iFGG, namely: 1. Types of offences involved; 2. Characteristics of the suspects to be tested; 3. Who has access to the data; 4. Data retention and use; and 5. Availability of a review mechanism. These factors all presuppose the preliminary safeguard identified in section 3.2 on iFGG as a method of last resort. It is incumbent upon law enforcement to show proof beforehand that they have utilized other less privacy intrusive methods, such as STR profiling, and are still left without a suspect lead before using iFGG. In other words, it would be disproportionate for law enforcement to use iFGG if criminal identification can be already be achieved using STR profile matching.

*First*, type of offences involved. In *Gaughran*, the applicable law limited the collection and retention of DNA data to recordable offences [36]. However, the ECtHR did not find such limitation sufficient but it did not specify further what types of offences are covered. Following ECtHR's reasoning in *Petrovic*, an appropriate safeguard for iFGG should be two-pronged: one is to limit it to violent crimes, and two is to specify what those violent crimes are. It is not sufficient to make a general indication that iFGG applies to all violent crimes [36]. For example, a recent development in Sweden, which takes effect in July 2025, allows iFGG use only for murder and aggravated rape [69]. Such a limitation creates the necessary balance in using iFGG which potentially exposes more sensitive data than STR-based methods as explained in section 3.1. A CoE Recommendation (cf. section 4.3) providing a list of crimes covered by iFGG at the CoE level will be a welcome development that will guide DTC-GT companies when they update their terms of agreement.

*Second*, characteristics of the suspects to be tested, such as criminal record, age and other special circumstances [22,41]. These characteristics refer to known individuals, i.e., their criminal records are known, including their past convictions and their age. In iFGG, the main concern of law enforcement is to identify the source of the forensic sample found at the crime scene (cf. section 5.1), i.e., iFGG will only be applied to traces and not to samples taken from suspects. Hence, a definition of the categories of suspects is not necessary.

*Third*, on who has access to the data. Safeguards related to data access revolve around the following: 1. Who requests the use of iFGG; 2. Who approves the request; and 3. Who handles iFGG data. *As to who requests the use of iFGG*, the burden falls on law enforcement in charge of the criminal investigation. They have to prove that they have exhausted other methods to no avail (cf. section 3.2). *As to who approves the request*, three possible authorities can be considered: a) the head of the law enforcement team in charge of the case; b) the prosecutor in charge of the case; and c) a judge. Given that SNP data are only meant to produce suspect investigational leads within criminal investigations, they are not meant to be directly used in court cases [2,4]. The approval of a judge or a court may not be deemed necessary by domestic authorities for iFGG to commence [9,10]. However, a more robust protection of these sensitive data should require the approval of a judge or a court prior to iFGG use. In Maryland, for example, the police has to obtain a warrant before conducting iFGG [15]. The pressure of solving a criminal case falls upon law enforcement, hence, there is a perceived bias that they will just approve iFGG in any event to speed up solving the case. Hence, it lends more objectivity to the process when the approving authority is outside the law enforcement system. *As to who handles iFGG data*, it should only be the law enforcement team in charge of the case. If external (technical) personnel is necessary, an appropriate safeguard is to allow them to handle data only when strictly necessary. All these safeguards, when put together, refer to limited use of data to specific and limited personnel and only when it is strictly necessary in fulfilling the purpose of criminal identification using iFGG.

*Fourth*, data retention and use. The SNP data used in iFGG come from forensic samples, hence, they are unknown until they are subsequently linked to a known individual. Based on the ECtHR's disposition in previous cases (cf. section 3.1), we argue that if the ECtHR limits the storage period of STR data, *a fortiori*, it will require more limited retention period for SNP data. Following the safeguard we proposed in section 5.2, SNP data should be destroyed once the criminal investigation is completed with finality.

*Fifth*, availability of a review mechanism. There should be a procedure that allow individuals to question the continued retention of their DNA data, preferably an “independent review” mechanism (para. 119) [22]. This mechanism is in keeping with the data protection principles of accuracy and data access [18,54,55,97,98]. In principle, this factor does not exactly apply to iFGG given that the SNP data generated do not pertain to known data subjects. It only applies once the individual source is identified. This review mechanism allows these known data subjects to request the deletion of their data once the purpose of the retention has been achieved. For a more robust protection of data privacy, it is not sufficient to assume that the people who have handled these data and are tasked to erase them, perform this duty, for example, due to human error, sloppiness, lack of interest and lack of sensitivity in protecting data privacy, time and cost involved, lack of technical know-how, conflict of interest, among others. Moreover, supervisory authorities should include the task of double-checking that these data have been erased in their regular audits of the institutions involved. This review mechanism also applies to the review of the whole iFGG process, whether law enforcement observed the appropriate safeguards or not, with the reviewing officer having the power to impose sanctions and penalties in case of breach, whose details are to be specified in the iFGG-enabling law (cf. section 4.3). What makes this review mechanism complicated is the question of allowing genetic relatives who can also be identified given the shared nature of DNA data [33,99]. We have dealt with this topic in a separate article, where we explained the current disposition of the ECtHR and a way to go forward (pp. 15–16) [13].

### 6.3 Safeguards related to the iFGG process

In this section, we propose safeguards that protect data privacy for each step of the iFGG process we previously illustrated and explained (cf. Fig in Ref. [4]). These safeguards revolve around general data protection principles, specifically data minimization, purpose limitation, accuracy and storage limitation [18,54,55,87]. A preliminary safeguard is required before discussing these steps: only competent law enforcement personnel should handle iFGG data. There should be an internal policy on the qualifications of personnel to be authorized to conduct iFGG, such as their knowledge of and adherence to data privacy principles, aside from their technical know-how.

The *first* step is the generation of SNP profile data from the forensic sample found at the crime scene [4]. This step presupposes that STR-based methods have been previously employed but they did not produce a suspect lead, in keeping with iFGG as a method of last resort (cf. section 3.2). The usual procedure is to generate more than 600,000 SNP markers. In 2023, QIAGEN released a new product that uses less than two percent of those markers (10,230 SNP markers) and they exclude “medically informative” SNPs, with the promise of providing investigative leads with “extended kinship associations” compared to STR systems [100,101]. We are aware that the conclusions of this study are relative to what the current technology can predict. What is important is that it should be able to produce investigational leads after STR-based methods failed to yield any. In any case, an appropriate safeguard is to use products or methods that require the least possible amount of SNP markers in iFGG—with proper scientific validation—that achieve the same purpose of generating suspect investigative leads in criminal investigations, in keeping with the data minimization principle.

The *second* step is the use of third-party genetic genealogy databases to generate matches. One possible complication is that the major databases—although they are mainly composed of individuals with European descent [102]—are based in the US. The decisions of the Court of Justice of the European Union (CJEU) in *Schrems I* [86] and *Schrems II* [85]—although they belong to another European supranational court and that they do not directly concern law enforcement use of DNA data—render the use of US-based genetic genealogy databases complicated [84,103]. A case in point: Although the Swedish Police Authority (SPA) has successfully identified the criminal perpetrator in the double-murder Linköping case, they were prevented from using iFGG further by the Swedish Authority for Privacy Protection (SAPP) [73]. The SAPP required a more solid legal basis for the transfer of data to US-based genetic genealogy companies: a “change of law” (p. 27) [73], which was passed in 2025 [[68], [69], [70]]. An appropriate safeguard in this regard is to limit the use of commercial genealogy databases to those located in CoE member states, or to companies outside the CoE that provide effective legal remedies to CoE data subjects in case of data breaches. For example, GEDmatch has a separate section for EU residents in its terms of service with regard to their “additional rights” under the GDPR [104]. As to whether such clauses are sufficient and applicable to CoE member states outside the EU deserves a full-blown study in itself. Suffice it to say for the purpose of this article that the third country where these genetic genealogy databases are located should provide an “appropriate level” of protection to European residents (art. 14 (2) [18]). Further study on this topic should also cover the meanings of appropriate, adequate or essentially equivalent protection and the various modes of international data transfers, among others [19,55,[85], [86], [87]], including the issue of data ownership in the context of these data transfers [105]. In any case, whatever accreditation is given to a third country should specifically allow these law enforcement searches and that CoE residents should have an effective legal recourse to question the processing and storage of their personal data [85,86,106]. A corollary safeguard is to allow law enforcement searches for iFGG only in certified commercial genetic databases. The certification requirements should be provided for in the iFGG-enabling law (cf. section 4.3) but should in any case include the following minimum requirements: use of scientifically-validated methods from procurement of samples to analysis of results, company rules specifically allowing law enforcement searches, users can opt-in and opt-out anytime, and law enforcement data is not accessible by other users. We acknowledge that the opt-in and opt-out procedure has its own flaws, especially since it also exposes one's genetic relatives to possible identification, which is at the very core of iFGG as exhibited in the Golden State Killer case [1,15,49]. We refer the reader to our previous article touching upon genetic relatives when law enforcement use genetic data [13]. For the purposes of this article, we opine that requiring an opt-in and opt-out system is better than allowing law enforcement access to all data in the third-party DNA database. Proportionality requires the removal or erasure of data obtained from database providers upon the fulfilment of the purpose of the law enforcement search, including law enforcement data in these databases, which prevents them from obtaining ownership rights over these law enforcement data. Each member state can follow its own rules on the persons responsible for these certifications and how they are enforced, which should be indicated in the iFGG-enabling law.

The *third* step is genealogy work. Given that this work involves a lot of sensitive data exposure, a key safeguard is to limit data access to the members of the law enforcement team in charge of solving the case. The technical details of this access—through authorizations and passwords, for example—may vary as data security techniques develop but they should be specified in implementing rules and regulations on the use of iFGG. As to the genealogy work itself when law enforcement lacks competent personnel, it should engage its certified national forensic institute, or only accredited experts registered in a national database of experts. Given that iFGG has developed through what is called “citizen science”—the engagement of the public in solving cases that involve science such as DNA data in the case of iFGG—the question of accreditation and certification can be very tricky [107,108]. In the US, for example, a self-taught genealogist has been engaged by law enforcement to do genealogy work but it was reported that she actively searched DNA profiles of those who opted out of law enforcement searches [15,109]. The iFGG-enabling law (cf. section 4.3) should include minimum requirements that meet both proficiency and ethical standards coupled with a mechanism that enforces these standards such as a technical-ethical committee or board. The latter can aid in the enforcement of these proficiency and ethical standards, impose disciplinary actions for violation of these standards, administer certification examinations, and build a database of certified genealogists accessible to law enforcement [110]. These requirements emphasize the safeguard of employing competent personnel throughout the iFGG process that assure data accuracy and respect for data privacy.

The *fourth* step is additional investigation and third-party (a.k.a. target, targeted or reference) testing. The iFGG process can theoretically end in the third step with a suspect lead. However, in the absence of a lead, additional investigation is needed. What may give rise to controversy is the third-party testing component of this iFGG step, which should not be confused with mass DNA screening [[111], [112], [113]]. In the Linköping case, more than 6000 men were asked to submit DNA samples for mass DNA screening, whereas only 15 volunteers were sufficient for third-party testing within iFGG [73]. Proportionality wise, third-party testing within iFGG appears to be more acceptable given that the sample size of new volunteers is more specific and far smaller, although obviously their numerous genetic cousins are implicated in the search and tree-building process, albeit not treated as suspects. At the same time, an obvious but important safeguard is called for: third-party testing should only be done when genealogy work does not yield suspect investigational leads. And the use of third-party testing should focus on specific individuals for the purpose of including or excluding branches of a family tree which is another way of limiting sample collection to the minimum.

### 6.4 Other factors

To complete the evaluation of iFGG, we discuss other factors that cannot be classified above but are relevant in evaluating the legitimacy of iFGG within the ECHR regime. We identified four factors.

*First*, there is a need to obtain a judicial or court approval prior to the use of iFGG. We discussed in section 6.2 that the approving authority for iFGG should be outside the law enforcement system. However, prior court approval is not commonly required in iFGG [20,73] mainly because iFGG data are currently not presented as evidence in criminal courts [4,50]. In short, the argument is that the judiciary is not yet engaged during the iFGG process. In the Netherlands, the public prosecution office opted to ask court approval to use iFGG in two pilot cases, and the presiding judge approved its use under current Dutch law [9,10], although we think that such legislation is insufficient [8,114]. In the Swedish case, which also involved a pilot study, we argue that it would have been better if a court approval was also granted prior to iFGG use in the Linköping case. It would have addressed the issues that led to the later decision to put on hold further iFGG use in the country, including possible objections from their data protection office [73]. We then propose as an appropriate safeguard that court approval be required—not merely an option, as in the case of the Netherlands—prior to iFGG use for a more robust protection of genetic data privacy. This makes iFGG use different from STR profiling that is currently done by law enforcement without prior court approval.

*Second*, we explained that access to iFGG data should only be confined to the law enforcement team handling the case with their corresponding safeguards (cf. Sections 6.2, 6.3). Regardless of the involvement of the prosecutor [20], all persons who have access to iFGG data should be spelled out in the iFGG-enabling law, together with their duties and responsibilities. An appropriate safeguard in this regard is the specification of possible penalties in case of breach, as for example, when there is an unlawful disclosure of personal data by authorized personnel, and unlawful access and/or disclosure of data by unauthorized personnel. These penalties will serve as a deterrent and a reminder of the sensitivity of the data involved. They also serve as a balancing measure on the part of the data subjects, i.e., they are assured that the exposure of their personal data for the resolution of crimes is only directed towards that purpose, and should there be a breach, the offenders will be subject to penalties as provided for in the iFGG-enabling law. A class action suit has been filed in the United States against the parent company of GEDmatch, Verogen (bought by Qiagen in 2023), accusing it of allowing law enforcement to access personal data of consumers who opted out of law enforcement searches from 2019 to 2023 [15,115,116]. The outcome of this case—albeit from another jurisdiction—will help provide benchmarks on the kinds of penalties that can be meted out on similar cases of data breaches.

*Third*, require law enforcement to conduct a Data Protection Impact Assessment (DPIA) prior to iFGG use. In the cases involving law enforcement use of DNA data (cf. section 3.1), there was no discussion on the need to conduct a DPIA. Those cases were decided under the CoE regime, whose relevant data protection law—the Data Protection Convention or its modernized version (Convention 108+) that is not yet in force—does not require a DPIA [18,54] compared to similar EU legislations, such as the Law Enforcement Directive (art. 27) [87] and the General Data Protection Regulation (art. 35) [55]. Given the potential exposure of more sensitive personal data via iFGG, we argue that a DPIA be made as a mandatory requirement in all member states and such DPIA be properly assessed and approved by a court. A practical guide—released more than 30 decades after the publication of the CoE Police Recommendation—recommends the making of a DPIA when using new data processing technologies (p. 9) [62]. A CoE-wide DPIA format can be included in the iFGG Recommendation proposed in section 4.3. The safeguards identified in this article can form part of this DPIA, including those that have been identified by other scholars (p. 5) [117].

*Fourth*, aside from the mainly scientific and legal concerns presented in this article, there have been some specifically ethical and social considerations on iFGG. These have been properly considered in various studies: bioethical perspectives [12,30,31], its relational [33] and social value [32] and the perception of some stakeholders [34]. They are mentioned here in order to provide a holistic picture of other issues surrounding iFGG, although they require further research. Following the case law of the ECtHR, a proper assessment of iFGG's legitimacy under the ECHR regime only requires that all the various aspects of the four-fold test with their corresponding safeguards are considered.

One final consideration in iFGG concerns the nature of the data generated by the method. As explained previously, the method only generates suspect investigational leads (cf. section 5.1), which are usually investigated further and confirmed using regular STR testing [2]. The latter can yield evidence, which can be presented before criminal courts. In this regard, we propose as a final safeguard to prohibit the use of suspect leads generated through iFGG as sole evidence for conviction. This safeguard is in keeping with the nature of the results of iFGG: that they are mere suspect investigational leads. Judges or juries should not be allowed to convict an individual based on DNA-based evidence alone. DNA evidence only shows that the genetic fingerprint of a particular individual is present in the crime scene, but that DNA sample could have been brought there through contamination or planting of DNA evidence. Hence, other corroborating evidence should also be presented such as but not limited to physical possibility of the suspect being present in the crime scene, intent and motive. This safeguard may appear obvious—as suspect leads are not usually presented in criminal courts—but it adds another layer of data privacy protection, i.e., the unnecessary exposure of SNP data in criminal courts. This safeguard does not prevent the criminal defense team from questioning the process of arriving at these suspect investigational leads via iFGG. However, the team will only be able to pursue that line if their client—the suspect—is first confirmed beyond the iFGG process [4]. Before the confirmatory step using STR testing and/or SNP through direct matching [4,118], there is no confirmed suspect to be brought before a criminal court to speak of.

A corollary safeguard is to require law enforcement as data controller to inform data subjects about the use of their personal data after the conclusion of the investigation. In order not to compromise the investigation, it is possible for law enforcement not to inform data subjects about the processing of their data. However, once the law enforcement purpose has been achieved, data subjects should be informed that they have been subject of data processing [62]. This safeguard will allow data subjects to question the appropriateness of their inclusion in the investigation and request a copy of their personal data, among others (cf. last paragraph of section 6.2 on the complex reality of genetic relatives of these data subjects). As earlier clarified, the use of DNA data in iFGG is an interference with the right to respect for private life of an individual (section 3). However, its processing is still allowed as long as it passes the tests of legality (section 4), legitimate purpose (section 5) and proportionality (section 6).

## 7 Conclusion

Although iFGG is a powerful tool to help solve cold cases, it has to stand on solid legal ground to avoid legal battles in the future and, particularly, to ensure that its application respects fundamental human rights, such as the right to privacy. The results of applying the ECtHR four-fold privacy test to iFGG in this article reveal that the legitimacy of iFGG within the ECHR regime is not that straightforward.

The preliminary interference test shows that the SNP profile data used in iFGG from whatever source is protected data under Article 8 ECHR. Hence, its use by law enforcement is an interference with the right to data privacy. As a preliminary safeguard, law enforcement should only use iFGG—which makes use of SNP data that potentially expose more sensitive data—if STR-based methods did not yield any suspect lead, i.e., a method of last resort. Under the lawfulness test, the creation of a CoE Recommendation on iFGG that will serve as a guide for domestic iFGG-enabling laws is crucial. This is in line with the ECtHR's ruling in *Petrovic* and the Swedish experience on using iFGG. Under the legitimate aim test, iFGG should be used for the immediate purpose of criminal identification but its mediate purpose—future criminal identification in unrelated cases—should be prohibited. We opine that the ECtHR will not allow the storage of the more sensitive SNP data once their purpose of identifying the source has been achieved, that is, once the SNP data are already linked to a known individual with finality.

Under the proportionality test, we identified several safeguards some of which are specific to iFGG. Unlike in the current STR-based method, prior judicial approval is necessary for the use of iFGG by law enforcement given that more sensitive data are used. A data protection impact assessment (DPIA) should accompany such judicial application where the safeguards identified in this article are applied to the case at hand. It has to be demonstrated that law enforcement makes use of the least number of SNP-markers possible—which means minimum exposure of sensitive data—that still allow effective criminal identification following scientifically validated methods. The use of iFGG should be limited to violent crimes specified both in its enabling law and in the terms and conditions of genetic genealogy databases. Genetic genealogy databases employed should be within the Council of Europe, countries duly-recognized as affording equivalent protection to CoE residents, or companies that afford effective legal remedies to CoE data subjects. Moreover, the use of SNP data—should it reach the court—as sole evidence for criminal conviction should be prohibited.

Law enforcement will always be confronted with a dilemma in its effort to solve crimes while respecting genetic data privacy. On the one hand, there is the urgent need to identify criminal perpetrators to bring them to justice, provide a closure to victims and their families, maintain peace and security in society, and prevent future crimes. On the other hand, there is the need to protect genetic data, which could expose not only the identity of its individual source but also sensitive traits such as medical information of the source's genetic family. This article is but one attempt to identify safeguards that help strike the balance between these two apparently opposing concerns so that the use of new technology—in this case, iFGG—will continue to respect the right to privacy enshrined under Article 8 ECHR while law enforcement fulfils its legitimate role of solving and preventing crimes. The numerous safeguards identified in this article should be understood not as onerous duties to be mechanically fulfilled but rather as positive duties that affirm and protect the right to genetic data privacy, even within criminal investigations. In that way, the dream of a safe and crime-free society is always balanced with respect for genetic data privacy.

## CRediT authorship contribution statement

**Oliver M. Tuazon:** Writing – review & editing, Writing – original draft, Resources, Methodology, Formal analysis, Conceptualization. **Bart Custers:** Writing – review & editing, Supervision, Resources, Methodology. **Gerrit-Jan Zwenne:** Writing – review & editing, Supervision, Resources, Methodology.

## Declaration of competing interest

The authors declare that they have no known competing financial interests or personal relationships that could have appeared to influence the work reported in this article.
